# Protocol for a multi-modal performance-profiling workflow of CAR T cell products

**DOI:** 10.1016/j.xpro.2026.104571

**Published:** 2026-05-15

**Authors:** Sarah Schulenberg, Michelle Loeser, Michael Schmueck-Henneresse

**Affiliations:** 1 Berlin Institute of Health (BIH) Center for Regenerative Therapies, Experimental Immunotherapy, BIH at Charité – Universitätsmedizin Berlin, Berlin 13353, Germany; 2 Ajmera Transplant Centre, University Health Network, Toronto, Canada; Department of Immunology, University of Toronto, Toronto, ON, Canada

**Keywords:** Cell-based Assays, Flow Cytometry, Cancer, Immunology

## Abstract

Functional characterization of engineered T cell products is essential for adoptive cell therapy development. Here, we present multi-modal profiling of CAR T cell products combining 33-marker full-spectrum flow cytometry (FSFC) with live-cell serial tumor-killing imaging. We describe steps for cell preparation, antibody staining, acquisition, serial killing assay, and integrated data analysis. This protocol is readily adaptable to other engineered T cell therapies.

For complete details on the use and execution of this protocol, please refer to Schulenberg et al.^1^

## Highlights

• Step-by-step protocol for 33-marker FSFC-based CAR T cell product profiling
• Sample preparation and spectral cytometry acquisition of T cell products
• Target-cell stimulation and live-cell serial tumor-killing imaging
• Integrated analysis of phenotypic and functional CAR T cell attributes

## Abstract (editor highlights)

Publisher’s note: Undertaking any experimental protocol requires adherence to local institutional guidelines for laboratory safety and ethics.

## Graphical abstract

**Figure:**
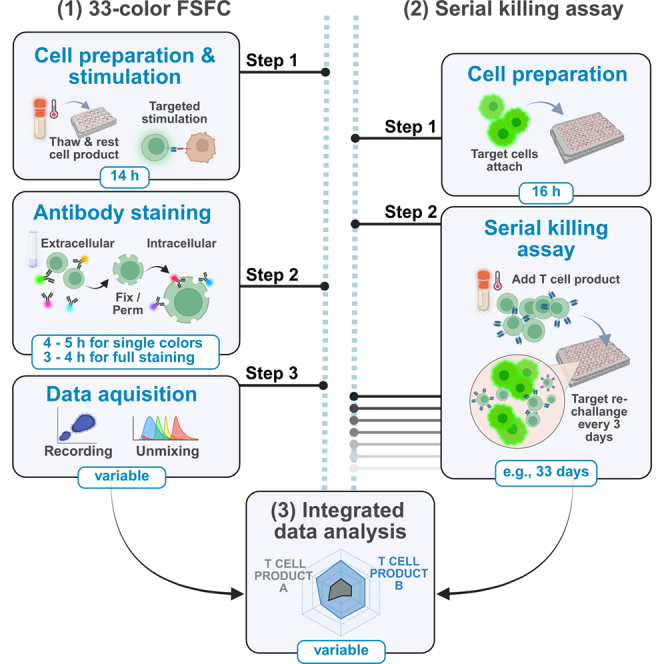

## Before you begin

Detailed characterization of therapeutic T cell products is essential for functional benchmarking in adoptive cell therapy.^2^^,^^3^ FSFC enables high-parameter protein-level analysis from limited sample material in a single measurement.^4^ By capturing large numbers of individual cellular events, FSFC provides a nuanced view of the phenotypic diversity within T cell products and supports contextualization of functional outcomes.^1^

The workflow was developed to characterize therapeutic T cells, with a specific focus on GD2.C7R CAR T cells.^5^ For this reason, markers detecting the GD2-CAR (14g2a scFv) and the C7R transgene (CD34 ectodomain) were included, which necessitates the use of GD2-positive target cells such as LAN-1. The panel incorporates markers defining T cell identity and major subsets (CD3, CD4, CD8), forming the foundation for downstream analyses. Prior to application in the CAR T cell setting, the panel was validated using peripheral blood mononuclear cells (PBMCs) and isolated T cells from healthy donors, which served as reference populations for optimizing staining conditions, gating strategies, and marker resolution.^1^

To capture differentiation and tissue-related features relevant to CAR T cell behavior, CD45RA and CCR7 classify memory states, while CD103 (tissue residency) and CXCR3 (infiltration and homing) provide information on tissue-homing potential. Additional markers assess activation (CD137, CD154, CD25, CD26, HLA-DR), effector function (IFN-γ, TNF-α), proliferation (Ki-67), and survival and susceptibility to activation-induced cell death (BCL-2, CD95), providing insight into recent stimulation, functional responsiveness, expansion potential, and cell fate after activation. Inhibitory receptors linked to T-cell regulation and exhaustion are captured by BTLA, CTLA-4, LAG-3, PD-1, TIM-3, and TIGIT. Cytokines (IL-2, IL-4, IL-17 A, IL-22) and chemokine receptors (CCR4, CCR6) further refine assessment of functional polarization and trafficking (Table 1). A detailed rationale for the selection and interpretation of these markers is provided in our accompanying research article,^4^ where the panel was applied to clinical CAR T cell products.

**Table 1:** Functional overview of markers included in the FSFC panel for CAR T-cell characterization

Marker category	Markers	Functional relevance in CAR T cells
T cell identity	CD3, CD4, CD8	Defines T cell lineage and major subsets
CAR/construct identity	CD34, 14G2a (1A7)	Identification of C7R and GD2-CAR in clinical T cell-products
Differentiation/memory	CD45RA, CCR7	Classification of naïve and memory states
Tissue localization	CD103, CXCR3	Tissue residency, infiltration, and homing
Activation	CD137, CD154, CD25, CD26, HLA-DR	Indicators of recent stimulation
Effector function	IFN-γ, TNF-α	Cytokine production and functional responsiveness
Proliferation	Ki-67	Cellular proliferation and expansion potential
Survival/apoptosis	BCL-2, CD95	Survival signaling and susceptibility to AICD
Inhibitory receptors	BTLA, CTLA-4, LAG-3, PD-1, TIM-3, TIGIT	Immune regulation and exhaustion
Functional polarization	IL-2, IL-4, IL-17 A, IL-22	Helper lineage polarization
Trafficking	CCR4, CCR6	Migration and tissue homing
Viability/QC	Live/Dead	Discrimination of live and dead cells to ensure accurate phenotypic and functional analysis

Finally, FSFC-derived phenotypic and functional data are integrated with image-based recording of serial tumor-cell killing. This combined workflow enables dynamic assessment of CAR T cell cytotoxicity and links phenotypic states to functional performance within the same experimental framework.

### Innovation

This protocol introduces methodological advances that extend current applications of FSFC and functional T cell assays. FSFC is commonly used to characterize T cell subsets within heterogeneous samples such as PBMCs,^4^ but it is less commonly applied for deep profiling a therapeutic T cell product. The protocol enables simultaneous detection of 33 markers within an enriched T cell compartment, allowing high-resolution phenotyping of engineered T cells at a depth rarely implemented for a single T cell population using FSFC. In addition, the workflow integrates FSFC-based phenotyping with quantitative killing assay readouts, providing a structured approach to link phenotypic states with functional performance. This combined strategy enables comprehensive functional benchmarking of CAR T cell products by directly relating differentiation, activation, and exhaustion profiles to cytotoxic capacity within the same experimental framework.^1^

### Institutional permissions

The clinical trial providing patient-derived CAR T cell products was approved by the Baylor College of Medicine Institutional Review Board and Biosafety Committee, and by the U.S. Food and Drug Administration (ClinicalTrials.gov identifier: NCT04099797). Written informed consent (and assent when applicable) was obtained for all patients, including consent for use of samples in research analyses. The study was conducted in accordance with the Declaration of Helsinki. All samples were de-identified before shipment and experimental use. Work with genetically modified T cells and human samples was performed in accordance with local biosafety and ethics regulations at Charité – Universitätsmedizin Berlin. The study involving healthy donor PBMCs was approved by the Ethics Committee of Charité – Universitätsmedizin Berlin (EA4/091/19).

### Technical considerations

FSFC panels must be adapted to the specific instrument on which they are run, as optical configurations, detector sensitivities, and overall spectral performance vary between devices. In this protocol, the panel was optimized on a Cytek Aurora. Although Cytek Aurora instruments use the same unmixing principles, differences in detector characteristics, optical alignment, and instrument configuration require validation on each individual cytometer. This setup includes generating single-stained controls for spectral unmixing and fluorescence-minus-one (FMO) controls for defining gating boundaries on critical markers. The antibody dilutions provided here were optimized for our system and may require adjustment on other instruments. Because staining intensity can vary between antibody lots, titration of each new lot is recommended to ensure consistent performance.

Fluorochrome-conjugated antibodies must be protected from light and stored at 4 °C to maintain fluorophore stability. Accurate detection of activation and exhaustion markers requires appropriate stimulation of target cells and controls, as many of these markers are inducible. Regular instrument quality control and calibration help maintain consistent spectral performance, and isotype controls may be included when needed to support interpretation of low-level signals.

### Sample preparation

This protocol requires human T cell samples prepared prior to initiating the profiling workflow. CAR T cells used in this protocol were generated according to previously published procedures^6^ and consist of GD2-C7R-engineered T cells.^5^ Users should obtain CAR T cell products manufactured using their standard activation, transduction, and expansion methods. Detailed CAR T manufacturing steps are not included in this protocol.

For panel establishment, optimization, and control conditions, PBMCs and ex vivo-expanded T cells from healthy donors may be used. Depending on the markers of interest, these cells may be left unstimulated or activated prior to staining. Activation can be achieved through stimulation with PMA/ionomycin, polyclonal activation using CD3/CD28 stimulation (e.g., antibody-coated beads or plate-bound antibodies), or short-term expansion to induce proliferation markers such as Ki-67 or to assess exhaustion-associated phenotypes. For CAR T cells, antigen-specific activation may be performed using target cells expressing the cognate antigen. For GD2-CAR products, we recommend using LAN-1 neuroblastoma cells as a robust GD2-positive stimulation source. In all cases where intracellular cytokines are analyzed, add brefeldin A (BFA) after 1 hour of stimulation to block protein secretion and allow accumulation of cytokines inside the cells.

For spectral flow cytometry, users should prepare at least 1 × 10^6^ viable cells per condition for the full multicolor panel. For single-color controls, a minimum of 1 × 10^5^ cells per stain is recommended to ensure adequate signal detection and reliable unmixing, with higher numbers required when targeting rare markers. All samples should exhibit a viability > 80% and be free of clumping prior to staining or functional assays. Additional material should be prepared if serial killing assays or parallel analyses are planned.

### Stimulating cells for single-stained controls

**Timing: 16–18 h**

Here we describe how cells can be stimulated to generate clear positive signals for intracellular cytokines, activation markers, and proliferation markers for the use as single-stained reference samples. Either polyclonal CD3/CD28 stimulation or PMA/ionomycin stimulation may be used.

1. Option A: Polyclonal stimulation.
  a. Coat wells of a 24-well plate with anti-CD3 and anti-CD28 antibodies:
    i. Dilute anti-CD3 (1 mg/mL) 1:1000 and anti-CD28 (1 mg/mL) 1:1000 in sterile water.
    ii. Add 500 μL per well and incubate ≥ 2 h at 37 °C, 5% CO_2_.
  b. Remove coating solution with a pipette and wash once with 1 mL 1 × PBS; do not allow wells to dry.
  c. Add 1 × 10^6^ cells in 2 mL medium (RPMI + 10% FBS) per coated well and culture at 37 °C, 5% CO_2_.
  d. After 1 h, add Brefeldin A (BFA) to a final concentration of 2 μg/mL to block cytokine secretion.
  e. Incubate for an additional 15 h (total stimulation time: 16 h).
  f. After stimulation, carefully transfer cells in 5 mL tubes and centrifuge at 400 × *g* for 10 min at 4°C.
  g. Remove the supernatant with a vacuum aspirator pump.
  h. Add 2 mL 1 x PBS to each tube and centrifuge at 400 × *g* for 10 min at 4°C.
  i. Remove the supernatant with a vacuum aspirator pump.
2. Option B: PMA/ionomycin stimulation.
  a. Resuspend PBMCs in medium (RPMI + 10% FBS).
  b. Aliquot 1 × 10^6^ cells in 500 μL per tube.
  c. Add stimulants:
    i. PMA final concentration: 2 ng/mL.
    ii. Ionomycin final concentration: 0.5 μg/mL.
  d. Mix gently and incubate at 37 °C, 5% CO_2_.
  e. After 1 h, add BFA to 2 μg/mL.
  f. Continue incubation for 15 h (total 16 h).
  g. Add 2 mL 1 x PBS to each tube.
  h. Centrifuge at 400 × *g* for 10 min at 4°C.
  i. Remove the supernatant with a vacuum aspirator pump.

***Note:*** Including an unstimulated control in parallel with stimulated single-stain controls is recommended. If stimulation induces strong expression of the marker of interest in all cells, adding a small proportion of unstimulated cells to the stimulated sample before staining can help ensure that both positive and negative populations are present. This facilitates gating, confirms assay performance, and improves interpretation of single-stain controls.

### Preparation of single-stained controls

**Timing: 4–5 h**

Here we describe how to prepare single color controls for unmixing FSFC samples. In case of activation markers, stimulated cells may be used to gain appropriate signals (see stimulating cells for single-stained controls).

***Note:*** Single-stained controls should ideally be prepared using the same cell type as those analyzed with the full panel to ensure optimal spectral matching. We used freshly isolated PBMCs from healthy donors to create spectral references. The single-stained controls should exhibit strong expression of the marker of interest to ensure high signal intensities for accurate spectral definition. If the signal intensity in the single-stained control used for unmixing is lower than the full-stained sample, the signal will be extrapolated, which can result in unmixing errors. Therefore, carefully select appropriate stimulated cells for activation or exhaustion markers, and CAR T cells for detection of CAR-specific markers.

***Note:*** The use of beads as reference controls can lead to shifts in emission spectra due to differences in antibody-fluorochrome binding properties between beads and cells.^7^ However, modern compensation beads are optimized to reduce these discrepancies. When these limitations are considered, bead-based single-stained controls represent a faster and less labor-intensive alternative to cell-based preparations. Keep in mind to always treat the beads the same way as the cells (washing, fixation, permeabilization).

3. Prepare the fixation/permeabilization solution and 1x permeabilization buffer according to the manufacturers protocol (eBioscience FoxP3/Transcription Factor Staining Buffer Set (Thermo Fisher Scientific)).
4. Distribute the cells/beads into 5 mL tubes:
  a. For cells, use a minimum of 1 × 10^5^ cells per single-stain.
  b. For beads, refer to the instructions of the manufacturer (e.g., UltraComp eBeads™ Compensation Beads).
5. Wash cells:
  a. Add 2 mL 1 × PBS to each sample.
  b. Perform centrifugation at 400 × *g* at 4°C for 10 min.
  c. Remove the supernatant with a vacuum aspirator pump.

***Note:*** For the Zombie viability dye single stain, add now the respective volume (1:4000) of the dye (pipet up and down) and 1 x PBS if needed to reach a final 100 μL staining volume.

6. For all other samples:
  a. Resuspend the cells/beads in 50 μL BD Horizon™ Brilliant Stain Buffer.
  b. Add 2 μl Fc block (Human TruStain FcX, Biolegend) to the cell suspension and incubate 5 min at 20–22°C.

***Note:*** The Fc block is optional but is recommended for PBMCs to prevent antibodies from binding nonspecifically via Fc receptors (mainly FcγR/CD16, CD32, CD64).

7. For extracellular marker, add the respective volume of the fluorochrome-coupled antibody (pipet up and down) and 1 × PBS if needed to reach a final 100 μL staining volume.
8. Incubate samples for at least 20 min at 4°C in the dark.

***Note:*** The Zombie viability dye single stain should be incubated at 37°C instead of 4°C.

9. Wash samples:
  a. Add 2 mL 1 × PBS to each sample.
  b. Perform centrifugation at 400 × *g* at 4°C for 10 min.
  c. Remove the supernatant with a vacuum aspirator pump.
  d. Loosen the pellet via vortexing.
10. Add 500 μL of the fixation/permeabilization solution to each sample and vortex.
11. Incubate samples for 30 min at 20–22°C in the dark.
12. After incubation, wash samples:
  a. Add 2 mL 1× permeabilization buffer to each tube.
  b. Centrifuge at 450 × *g* for 10 min.
  c. Remove the supernatant with a vacuum aspirator pump.
  d. Loosen cell pellet via vortexing.
13. For intracellular marker, add the respective volume of the fluorochrome-coupled antibody (pipet up and down) and 1 × permeabilization buffer if needed to the cell suspension to reach 100 μL staining volume.
14. Incubate 30 min at 4°C in the dark.
15. Wash samples:
  a. Add 2 mL 1 × PBS to each sample.
  b. Perform centrifugation at 450 × *g* at 4°C for 10 min.
  c. Remove the supernatant with a vacuum aspirator pump.
  d. Loosen the pellet via vortexing.
16. Resuspend the cells in 200 μL 1× permeabilization buffer and measure samples at an FSFC-device.

***Note:*** Single-stained controls can also be prepared in 96-well plates by reducing the standard 2 mL wash volume used in all washing steps to 200 μL. When using reduced volumes, an additional centrifugation step may be helpful to ensure efficient removal of residuals and to improve pellet stability.

### Thawing CAR T cells

**Timing: 2–18 h**

Here we describe how to thaw cryopreserved CAR T cell products prior to full cell staining. While this workflow is compatible with both freshly isolated and cryopreserved samples, the latter require thawing and a resting period before staining, as outlined below.

17. Thaw cryopreserved cells rapidly in a 37 °C water bath until only a small ice crystal remains.
18. Transfer cells dropwise into 10 mL prewarmed complete medium (e.g., RPMI + 10% FBS).
19. Centrifuge at 400 × *g* for 10 min at 20–22°C.
20. Remove the supernatant with a vacuum aspirator pump.
21. Resuspend cells in fresh complete medium and rest for at least 2 h or 18 h at 37 °C, 5% CO_2_.

### Antigen-specific stimulation with target cells

**Timing: 12–14 h**

Here we describe how to stimulate CAR T cell products with antigen-expressing cells to assess CAR-mediated activation and identify phenotypic changes following target engagement. For GD2-CAR T cells, LAN-1 neuroblastoma cells serve as a robust GD2-positive stimulation source.

22. Combine CAR T cells and LAN-1 cells at a 1:1 ratio in complete medium (e.g., RPMI + 10% FBS) in 2 mL medium per well of a 24-well plate.
23. Culture at 37 °C, 5% CO_2_.
24. After 1 h, add BFA to 2 μg/mL.
25. Continue incubation for 11 h (total 12 h) to induce antigen specific activation.
26. Gently resuspend the coculture and transfer to 5 mL tubes.
27. Centrifuge at 400 × *g* for 10 min at 20–22°C.
28. Remove the supernatant with a vacuum aspirator pump.
29. Add 2 mL 1 × PBS to each tube.
30. Centrifuge at 400 × *g* for 10 min at 4°C.
31. Remove the supernatant with a vacuum aspirator pump.

***Note:*** Including an unstimulated CAR T cell control in parallel is recommended. If activation is strong and all cells become positive for a given marker, mixing a small proportion of unstimulated cells into the stimulated sample before staining can help ensure both positive and negative populations are present for gating.

## Key resources table

**Table:** 

REAGENT or RESOURCE	SOURCE	IDENTIFIER
**Antibodies**		

Spark UV387 anti-human CD45RA, clone HI100 (1:200)	Biolegend	Cat#304179
Mouse Anti-Human CD25.Star Bright UltraViolet 445, clone MEM-181 (1:100)	BioRad	Cat#MCA2127SBUV445
BUV563 mouse anti-human CD26, clone M-A261 (1:200)	BD Biosciences	Cat#749318
BUV661 Mouse anti-human CD103, clone Ber-ACT8 (1:400)	BD Biosciences	Cat#749993
Spark YG 593 anti-human CD4, clone SK3 (1:50)	Biolegend	Cat#344672
BUV805 mouse anti-human CD8, clone RPA-T8 (1:200)	BD Biosciences	Cat#749366
Brilliant Violet 421 anti-human CD197 (CCR7), clone G043H7 (1:50)	Biolegend	Cat# 353208
Anti-human 14g2a, clone 1A7 (1:200)	Provided by Bilal A. Omer (Center for Cell and Gene Therapy, Texas Children’s Hospital, Houston Methodist Hospital, Baylor College of Medicine, Houston, TX, USA; Texas Children’s Cancer and Hematology Centers, Texas Children’s Hospital, Baylor College of Medicine, Houston, TX, USA)	N/A
BV480 Mouse anti-human CD34, clone 563 (1:400)	BD Biosciences	Cat#746415
Brilliant Violet 605 anti-human CD152 (CTLA-4), clone BNI3 (1:50)	Biolegend	Cat#369610
Brilliant Violet 650 anti-human CD366 (Tim-3), clone F38-2E2 (1:20)	Biolegend	Cat#345028
Brilliant Violet 711 anti-human CD196 (CCR6), clone G034E3 (1:200)	Biolegend	Cat#353436
Brilliant Violet 785 anti-human CD279 (PD-1), clone EH12.2H7 (1:100)	Biolegend	Cat#329930
Spark Blue 550 anti-human CD3, clone SK7 (1:50)	Biolegend	Cat#344852
PE/Dazzle 594 anti-human CD272 (BTLA), clone MIH26 (1:400)	Biolegend	Cat#344522
PE/Fire640 anti-human TIGIT (VSTM3), clone A15153G (1:400)	Biolegend	Cat#372744
PE/Fire700 anti-human CD194 (CCR4), clone L291H4 (1:400)	Biolegend	Cat#359436
PE/Fire810 anti-human HLA-DR, clone L243 (1:400)	Biolegend	Cat#307683
Alexa Fluor 647 anti-human CD95 (Fas), clone DX2 (1:200)	Biolegend	Cat#305618
APC/Fire810 anti-human CD183 (CXCR3), clone G025H7 (1:100)	Biolegend	Cat#353762
Alexa Fluor 488 anti-human IL-4, clone MP4-25D2 (1:50)	Biolegend	Cat#500817
APC/Fire750 anti-human INF-g, clone 4 S.B3 (1:400)	Biolegend	Cat#502548
BUV496 Mouse Anti-Human CD40L (CD154), clone 24-31 (1:50)	BD Biosciences	Cat# 752853
BUV737 Rat anti-human IL-2, clone MQ1-17H12 (1:100)	BD Biosciences	Cat#612836
Brilliant Violet 570 anti-human IL-17 A, clone BL168 (1:200)	Biolegend	Cat#512324
Brilliant Violet 750 anti-human Ki-67, clone ki-67 (1:800)	Biolegend	Cat#350536
NovaFluor Blue 660-120 S anti-human CD223 (LAG-3), clone 3DS223H (1:200)	Thermo Fisher Scientific	Cat#H048T03B08
PacificBlue anti-human/mouse Granzyme B Recombinant, clone QA16A02 (1:20)	Biolegend	Cat# 372218
PE anti-Bcl-2, clone 100 (1:200)	Biolegend	Cat#658708
PE/Cyanine5 anti-human CD137 (4-1BB), clone 4B4-1 (1:100)	Biolegend	Cat#309808
PE-Cy7 Mouse Anti-Human IL-22, clone MH22B2 (1:800)	BD Biosciences	Cat#567579
PerCP anti-human TNF-a, clone MAb11 (1:50)	Biolegend	Cat#502924
CD3 Monoclonal Antibody (OKT3)	eBioscience™	Cat#14-0037-82
Purified anti-human CD28 Antibody (CD28.2)	Biolegend	Cat#302902

**Biological samples**		

Patient-derived autologous (C7R) GD2.CAR T-cell products	Lin et al.^6^	ClinicalTrials.gov identifier: NCT04099797

**Chemicals, peptides, and recombinant proteins**		

BD Horizon™ Brilliant Stain Buffer	BD Biosciences	Cat#563794
BD Horizon™ Brilliant Stain Buffer Plus	BD Biosciences	Cat#566385
Human TruStain FcX	Biolegend	Cat#422302
Zombie NIR Fixable Viability Kit	Biolegend	Cat#423106
eBioscience™ Foxp3/Transcription Factor Staining Buffer Set	Invitrogen	Cat#00-5523-00
Ultracomp eBeads Compensation-Beads	Invitrogen	Cat#01-2222-42
Brefeldin A	Sigma-Aldrich	Cat#B7651-25MG
PMA	Sigma-Aldrich	Cat#P1585
Ionomycin	Sigma-Aldrich	Cat#I0634-5MG
EDTA, 0,5 M solution	VWR	Cat#E177-100ML
Bovine Serum Albumin	Sigma-Aldrich	Cat#A9418-10G
DPBS (1x)	Gibco, Thermo Fisher Scientific	Cat#14190-094
FBS	Sigma-Aldrich	Cat#F7524
RPMI 1640	Pan Biotech	Cat#P04-18525
Ampuwa® water	Fresenius Kabi AG	Cat#B230673

**Critical commercial assays**		

Alexa Fluor 700 Conjugation Kit (Fast) - Lightning-Link	Abcam	Cat#ab269824

**Experimental models: Cell lines**		

LAN-1 cells (WT)	Provided by Bilal A. Omer (Center for Cell and Gene Therapy, Texas Children’s Hospital, Houston Methodist Hospital, Baylor College of Medicine, Houston, TX, USA; Texas Children’s Cancer and Hematology Centers, Texas Children’s Hospital, Baylor College of Medicine, Houston, TX, USA)	N/A
LAN-1-GFP cel	Provided by Bilal A. Omer (Center for Cell and Gene Therapy, Texas Children’s Hospital, Houston Methodist Hospital, Baylor College of Medicine, Houston, TX, USA; Texas Children’s Cancer and Hematology Centers, Texas Children’s Hospital, Baylor College of Medicine, Houston, TX, USA)	N/A

**Software and algorithms**		

CellReporterXpress software	Molecular Devices	RRID:SCR_025681
SpectroFlo software, v3.2.1	Cytek	RRID:SCR_025494
FlowJo, v10.9.0	BD Biosciences	RRID:SCR_008520
OMIQ, accessed in 2024	Dotmatics	app.omiq.ai
GraphPad Prism, version 9	GraphPad Software	RRID:SCR_002798
R Statistical Software, v4.4.0	R Core Team	RRID:SCR_001905

## Step-by-step method details

This section describes a three part workflow consisting of: (1) FSFC-based staining and acquisition of a 33-marker panel to assess activation and phenotypic characteristics, (2) a serial killing assay to quantify sustained cytotoxic activity through repeated rounds of target-cell engagement, and (3) integrated analyses of flow cytometry and killing assay data to generate a combined functional profile of CAR T cell products.

### 33-color FSFC measurement

**Timing: 3–4 h (depending on sample number)**

Here we describe the staining of CAR T cell samples with the 33-color FSFC panel.

***Note:*** To measure cells stained with all antibodies in the panel, spectral references are required for each fluorochrome used (see Preparation of single stained controls). For this purpose, single-stained cells, or beads in the case of weak expression or rare populations, are measured on the spectral flow cytometer prior to the acquisition of the full stained samples. With the help of the individual emission spectra of the markers, the fully stained samples can be correctly unmixed. Reference controls should ideally be recorded each time a sample is acquired. However, reference controls can be saved and reused for a certain time. Always acquire new reference controls if there are changes in the instrument settings or reagents (e.g. antibody lot).

**CRITICAL:** Always measure unstained controls of each sample to assess the intrinsic emission spectra of the cells under investigation and to account for cellular autofluorescence.

***Note:*** For gating, record fluorescence-minus-one (FMO) controls. These should be prepared by following the full staining protocol while omitting one marker per sample, allowing accurate assessment of background fluorescence and proper gate setting.

1. Count the alive CAR T cells after washing (e.g., using the LUNA-FX7 Automated Cell Counter by Logos Biosystems for determining living cells) and resuspend with 1 × PBS.
2. Distribute 100 μL of cell suspension to 5 mL tubes per sample to be stained.

***Note:*** Use at least 1 × 10^6^ alive cells per full-stained sample to obtain a sufficient signal for each marker.

3. Add Zombie viability dye in a 1:4000 dilution to the cells.
4. Incubate them in the dark for 20 min at 37°C.
5. Wash cells:
  a. Add 2 mL PBS to each sample.
  b. Centrifuge at 400 × *g* at 4°C for 10 min.
  c. Remove the supernatant with a vacuum aspirator pump to leave approximately 50 μL residual volume (assessed by comparison to a reference tube containing 50 μL measured via pipette).
6. Prepare the master mix for extracellular staining:
  a. Add 50 μL BD Horizon™ Brilliant Stain Buffer (or 10 μL BD Horizon™ Brilliant Stain Buffer Plus) per sample to be stained.
  b. Add the volumes of all extracellularly stained antibodies (see key resources table and Schulenberg et al. (2025)^1^).

***Note:*** Antibody mastermixes may be centrifuged at high speed prior to use to remove potential aggregates. In our workflow, mastermixes are centrifuged at 14,000 × *g* for 5 min, and the supernatant is used for staining.

***Note:*** Brilliant Stain Buffer should be included for all samples stained with Brilliant Violet/Brilliant Ultraviolet fluorophores to minimize dye-dye interactions and spreading error.

***Note:*** Some antibody-fluorophore conjugates require additional or specialized buffers for optimal performance (e.g., NovaFluor antibodies require the dedicated NovaFluor Stabilization Buffer). Always consult the manufacturer’s instructions and incorporate any required buffer volumes into your total staining volume calculation.

7. Add the master mix to the cell suspension in 5 mL tubes (pipet up and down).
8. Incubate at 4°C in the dark for 20 min.
9. Wash cells:
  a. Add 2 mL PBS to each sample.
  b. Centrifuge at 400 × *g* for 10 min.
  c. Remove the supernatant with a vacuum aspirator pump.
  d. Loosen cell pellet via vortexing.
10. Add 500 μL of the fixation/permeabilization solution to each tube.
11. Vortex samples.
12. Incubate the samples for 30 min at 20–22°C in the dark.
13. Wash cells:
  a. Add 2 mL 1× permeabilization buffer to each tube.
  b. Centrifuge at 450 × *g* for 10 min.
  c. Remove the supernatant with a vacuum aspirator pump.
  d. Loosen cell pellet via vortexing.
14. Adjust the volume of the samples to 50 μL with 1× permeabilization buffer (estimated by visual comparison to a reference tube containing 50 μL measured by pipette).
15. Prepare intracellular staining master mix:
  a. Add 50 μL of BD Horizon™ Brilliant Stain Buffer (or 10 μL BD Horizon™ Brilliant Stain Buffer Plus) per sample to be stained.
  b. Add the volumes of all intracellularly stained antibodies (see key resources table and Schulenberg et al. (2025)^1^).
  c. Centrifuge master mix.

***Note:*** Depending on the antibody concentration determined during titration, the final staining volume in the full stain may exceed the recommended 100 μL. If this occurs, the staining volume should be adjusted, the cell suspension volume reduced, or a more concentrated staining buffer (e.g. BD Horizon™ Brilliant Stain Buffer Plus) considered.

16. Add intracellular staining master mix to the cells, incubate for 30 min at 4°C in the dark.
17. Wash cells:
  a. Add 2 mL 1× permeabilization buffer to each tube.
  b. Centrifuge at 450 × *g* for 10 min.
  c. Remove the supernatant with a vacuum aspirator pump.
  d. Loosen cell pellet via vortexing.
18. Resuspend the cells in 200 μL 1× permeabilization buffer and acquire samples on an FSFC-instrument.

***Note:*** If samples appear sticky (e.g., after stimulation with tumor cells), an alternative measurement buffer can be used to reduce cell aggregation and improve flow.

**Table:** Alternative measurement buffer

Reagent	Final concentration
PBS	base
Bovine Serum Albumin	0.5 %
EDTA	2 mM

### Setup and acquisition of the spectral flow cytometer

**Timing: 30 min setup, 1–8 h acquisition (depending on sample number), 30 min to several hours for unmixing samples**

Here we describe data acquisition on the Cytek Aurora spectral analyzer, covering experiment setup and acquisition of both reference controls and fully stained samples. For further guidance on instrument setup, refer to the Cytek Aurora reference guide (available at https://cytekbio.com/pages/user-guides).

**CRITICAL:** Ensure daily QC has been performed and passed on the instrument.

***Note:*** Always resuspend and filter cells directly prior to acquisition to avoid clogging the fluidics.

19. Experiment setup.
  a. Start the Cytek Aurora and the SpectroFlo software.
  b. Create a new experiment:
    i. Select all fluorochromes used in the panel.
    ii. Add a reference group for all the single-stained controls and select if beads or cells were used.
    iii. Add groups for samples and name them accordingly.
    iv. Create labels for each fluorescent tag according to the detected marker (e.g., Spark Blue 550 as CD3).
    v. Define acquisition settings for reference controls and samples.
20. Save and open the experiment.
21. Sample acquisition:
  a. Acquire reference controls (unstained, single-stained, and FMO samples):
    i. Adjust SSC and FSC gains for optimal scaling and signal range.
  b. Perform unmixing:
    i. Gate on target cell population or beads.
    ii. Identify positive and negative populations in the detector with the highest emission peak.
    iii. Verify that fluorochrome spectra of your reference controls match the theoretical emission spectra.
  c. Assess unmixing quality using single-stained controls.
    ***Note:*** Unmixing can be performed during sample acquisition or afterwards. Depending on the complexity of the panel, initial unmixing can be time-consuming. For panel establishment, additional quality assessment is advisable.
  d. If unmixing is satisfactory, acquire fully stained samples.
  e. Save and export FCS files for data analysis.

### Serial killing assay

**Timing: 33 days**

Here we describe how to perform a serial killing assay using the same unstained CAR T cells together with the corresponding antigen-expressing tumor targets. By assessing cytotoxic capacity, persistence, and functional robustness under repeated antigen exposure, this assay complements the phenotypic FSFC panel data to yield a comprehensive functional characterization of CAR T cell products.

**CRITICAL:** Select a tumor cell line that expresses both the target antigen recognized by the CAR T cells under investigation and a fluorescent reporter that enables quantification of tumor cell numbers over time. For GD2-CAR T cells, we use the GD2-positive LAN-1 neuroblastoma cell line constitutively expressing green fluorescent protein (GFP). Ensure that tumor cells are maintained under optimal culture conditions and exhibit high viability prior to initiating the assay, as poor tumor cell health can compromise assay performance and data interpretation.

**CRITICAL:** Measure samples in duplicates or triplicates to obtain robust data. Always include controls, including CAR T cells without tumor cells and tumor cells without CAR T cells.

22. Count alive tumor cells (e.g., via the LUNA-FX7 Automated Cell Counter (Logos Biosystems)).
23. Plate tumor cells in a 96-well clear-bottom imaging plate in cell culture medium, 100 μL per well.

***Note:*** We recommend using 4 × 10^5^ alive tumor cells per well. The optimal number of tumor cells depends on tumor cell size and the seeding density required to ensure both adequate cell health and image quality. The culture medium that supports both tumor cells and CAR T cells, should be empirically optimized in preliminary experiments. In our setup, LAN-1 tumor cells and CAR T cells were co-cultured in CTL medium. As LAN-1 cells are adherent, we plated them 4 h before adding T cells to allow adherence to the well bottom.

24. Add 100 μL of CAR T cell product at different effector-to-target ratios to each well with tumor cells.
25. As a control, leave one condition with tumor cells only.
26. If available, add 100 μL of non-CAR expressing T cells at different effector-to-target ratios to each well with tumor cells.

***Note:*** We applied effector-to-target (E:T) ratios of 2:1, 1:1, 1:2, and 1:6 to investigate killing differences. We recommend using duplicates for each condition.

27. Culture the plate at 37°C and 5% CO2 in a live-cell imaging system (e.g., the ImageXpress Pico Automated Cell Imaging System from Molecular Devices).
28. Set up continuous imaging to detect live tumor cells in each well.

***Note:*** E.g.: If you use GFP^+^ tumor cells, measure the total GFP area every 7 h.

29. Every three days, carefully remove 100 μL supernatant from each well and add 100 μL fresh tumor cells at the same cell number used for the initial seeding.
30. Ideally, keep imaging, until no further killing of tumor cells by CAR T cell products is observed (e.g., 33 days).

### Data analysis

**Timing: Variable**

Here we summarize key steps for data analysis. The full analysis workflow is described in detail in Schulenberg et al. (2025).^1^

31. FSFC data are unmixed in SpectroFlo (Cytek) and visually inspected to confirm correct unmixing.
32. Resulting FCS files are imported into FlowJo or OMIQ where conventional manual gating can be performed.

***Note:*** Other FCS-compatible platforms may be used if they support comparable workflows.

**Figure 1:**
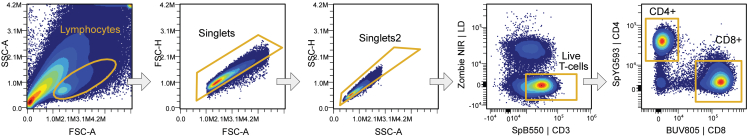
Representative gating strategy Example gating sequence used to define the T-cell population serving as input for high-dimensional analysis. Forward scatter (FSC) versus side scatter (SSC) plot illustrating the initial lymphocyte gate. FSC-area versus FSC-height plot used to select singlets and exclude aggregates. SSC-area versus SSC-height plot showing a second singlet gate to further refine the population. Viability and lineage gating using Zombie NIR Fixable Viability Dye and CD3 to define live T cells. CD4 versus CD8 plot displaying discrimination of CD4^+^ and CD8^+^ T-cell subsets. Percentages are omitted because the figure is intended to illustrate the gating workflow rather than representative subset frequencies.

***Note:*** For the CAR T cell analysis, both unstimulated and stimulated samples can be included.

***Note:*** CAR-specific markers may be excluded from clustering to emphasize the overall composition of the T cell product.

***Note:*** In stimulated samples, CAR detection can be reduced due to antigen engagement during co-culture, depending on the CAR construct and the antibody used for detection. Users may consider alternative CAR-binding reagents if specific CAR-level resolution is required.

33. Automated data cleaning can be applied using FlowCut, although alternative tools can be used based on user preference.
34. For high-parameter analyses, appropriate scaling of marker expression is essential. An arcsinh transformation can be applied in OMIQ, though other transformations (e.g., biexponential) are also suitable.
35. Dimensionality reduction can be performed using UMAP due to its ability to preserve global structure, but other methods (e.g., t-SNE) can be used depending on the analytical goals.
36. FlowSOM can be used for clustering, with other cytometry-compatible clustering algorithms being equally applicable.
  a. The starting population is defined as manually gated single, live T cells (Figure 1).

37. Visualizations and statistical analyses can be performed in R, although other statistical environments can be used.
38. Cluster abundances can be compared across sample groups of interest.
39. Relative marker expression within these clusters can be used to identify distinguishing markers, and corresponding cell populations can subsequently be isolated in each product using conventional manual gating, illustrating how high-dimensional analysis can be combined with classical gating for targeted validation of identified populations.
40. For each product, the frequencies of gated populations can be visualized in spider charts with chart axis labels reflecting biological interpretations of the marker combinations derived from the response-associated clusters.
41. Spider charts can incorporate a measures from the serial killing assays, based on the GFP-positive area over time as a surrogate for remaining tumor cells.
  a. CAR T cell performance can be calculated as the percentage of tumor-cell killing relative to the first time point.
  b. Overall serial-killing capacity can be quantified by integrating killing values across all rounds into a single performance score per product, with higher values indicating stronger killing.
42. FSFC-derived data points and integrative serial-killing values can be normalized for visualization, such that a larger inner area in the spider chart reflects better functional performance.

## Expected outcomes

Execution of this protocol is expected to produce spectral flow cytometry data of sufficient quality for downstream high-dimensional analysis. After unmixing and import, the gating sequence shown in Figure 1 should yield a clearly defined lymphocyte population, removal of doublets, and a stable live CD3^+^ T cell population with distinguishable CD4^+^ and CD8^+^ subsets. These gated events form the basis for subsequent computational steps.

**Figure 2:**
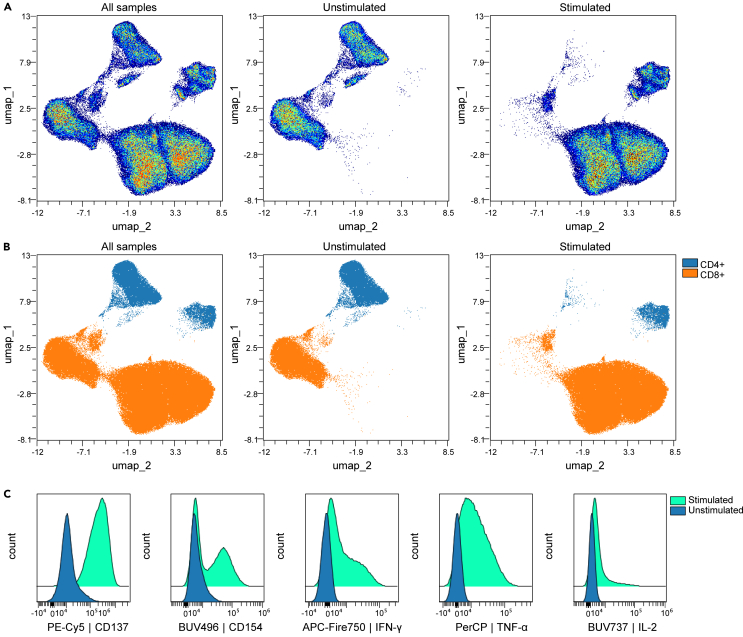
UMAP embeddings and stimulation-dependent marker differences (A) UMAP embeddings from two representative CAR T cell products. Left: Combined visualization of both samples to illustrate overall data structure. Middle: Unstimulated sample. Right: Stimulated sample. (B) Overlay of manually gated CD4^+^ and CD8^+^ T cell populations on the same UMAP embeddings. (C) Histogram overlays of selected activation markers comparing unstimulated and stimulated conditions from the same representative product.

Dimensionality-reduction methods such as UMAP should generate reproducible low-dimensional embeddings (Figure 2A). Unstimulated and stimulated samples typically occupy distinct regions within the embedding, reflecting stimulation-dependent phenotypic changes. Overlaying manually gated CD4^+^ and CD8^+^ subsets (Figure 2B) provide a straightforward check that major T cell lineages map consistently within the reduced-dimensional space. Histogram overlays of activation markers (Figure 2C) should show increased expression after stimulation, indicating effective activation and appropriate staining performance.

**Figure 3:**
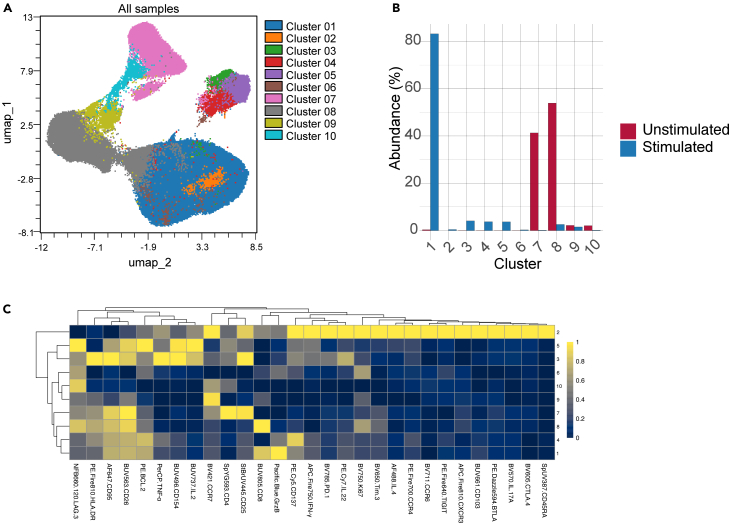
FlowSOM clustering and marker expression patterns on UMAP-derived data (A) FlowSOM clustering (k = 10) applied to the UMAP-embedded live T cell population from the representative samples shown in Figure 2. Each color represents a distinct cluster, illustrating the phenotypic granularity captured by the workflow. Cluster labels are not assigned because the figure is intended to illustrate the analysis workflow rather than dataset-specific biological annotation. (B) Comparison of cluster abundance between unstimulated and stimulated conditions for the same samples, shown as bar graphs. (C) Heatmap (min-max scaled) showing median expression of FSFC markers across the ten FlowSOM clusters.

FlowSOM clustering applied to the UMAP-embedded T-cell population generally results in a set of phenotypically distinct clusters (Figure 3A). The number and composition of clusters may vary depending on the dataset, but clusters typically show internally coherent marker-expression patterns. Cluster-abundance plots comparing unstimulated and stimulated conditions (Figure 3B) illustrate visualization of relative frequencies across conditions without reference to clinical outcome. Marker-expression heatmaps (Figure 3C) provide an overview of how markers are distributed across clusters and can help users assess whether the clustering output aligns with expected phenotypic patterns for their specific dataset.

The serial killing assay is expected to produce time-resolved measurements of GFP-positive target-cell area for each CAR T cell product. When normalized to the initial time point, these measurements typically show a decrease in GFP-positive area over time, reflecting target-cell elimination. From these data, an integrated killing value can be calculated for each product, for example by summarizing the area under the curve or by applying a predefined scoring approach. The detailed implementation of this assay is described in Schulenberg et al. (2025).^1^

The killing values can be combined with phenotypic readouts from the FSFC panel to generate spider-chart visualizations. For this purpose, both the FSFC-derived parameters and the integrated killing values can be normalized to a common scale. In these charts, larger relative areas correspond to higher values for the included parameters. The resulting plots provide an overview of how phenotypic and functional characteristics relate to one another across products. Additional examples of these integrated visualizations are provided in Schulenberg et al. (2025).^1^

## Limitations

Flow-cytometric phenotyping and killing assays have methodological limitations. In FSFC, panel design depends on the availability of suitable antibody clones and fluorophores. Many antibodies rely on tandem dyes engineered to occupy specific spectral regions for highly multiplexed panels. Due to their composition, tandem dyes are rather unstable and can be sensitive to experimental conditions (e.g., light exposure, fixation procedures, buffer composition), which can lead to degradation or decoupling, ultimately affecting emission profiles and marker resolution. Despite standardized acquisition settings, technical variability may arise when reference controls are acquired on different days or when panels are run on different devices, resulting in batch effects. Certain epitopes (e.g., chemokine receptors) are particularly sensitive to experimental conditions and may show reduced detection at low temperatures or after extended handling. Similar considerations apply to frozen samples, which can affect viability and the detection of sensitive epitopes. Finally, any phenotyping panel captures only a selected snapshot of markers and may not encompass functional or phenotypic states.

The killing assay likewise reflects a controlled experimental system. The defined time frame provides a reproducible window for assessing cytotoxicity, but it cannot fully capture the dynamic nature of in vivo interactions. Effector-to-target ratios are experimentally set to allow standardized comparisons, even though they may not mirror physiological conditions. These considerations are inherent to the method and can be managed through consistent experimental design and careful optimization. As the serial killing assay extends over several days, the T cell compartment may undergo dynamic changes during repeated antigen exposure, complementing the phenotypic snapshot obtained from the FSFC panel.

## Troubleshooting

### Problem 1

Marker signal too dim or absent (e.g., during the Preparation of single stained controls).

### Potential solution

Weak or missing marker signals may arise from insufficient antibody concentration, low-brightness fluorochromes, epitope masking during fixation and permeabilization, or rapid internalization of certain surface markers. Titrate each antibody to determine the optimal concentration, extend staining time or stain at 20-22°C and verify that the fixation/permeabilization protocol preserves the relevant epitopes. If needed, switch to a brighter fluorochrome or a more robust antibody clone.

### Problem 2

Poor unmixing or spillover artifacts.

### Potential solution

Spectral unmixing may fail when single-stain controls are too dim, do not match the experimental samples, or when fluorochromes are unstable or degraded. This results in over- or under-compensation and distorted marker expression. Ensure that each single-stain control is at least as bright as the fully stained sample and acquired under identical settings. Use antibody-capture beads or well-stained cells for single stains and recalculate the unmixing matrix before analyzing experimental data.

### Problem 3

Low chemokine receptor staining (e.g., during the Preparation of single stained controls).

### Potential solution

Chemokine receptors (e.g., CXCR3, CCR6, CCR7) are highly sensitive to freezing, handling, and activation. They internalize quickly and may be lost during prolonged staining or cold temperatures. Use fresh or gently cryopreserved samples and allow thawed cells to rest (e.g., 14-18 hours) to reduce stress-induced internalization. Reduce activation or handling times before staining. To improve surface detection, chemokine receptors can be stained at 20-22°C.

### Problem 4

Low T cell activation (e.g., stimulating cells for single-stained controls).

### Potential solution

Poor activation can result from stressed or damaged cells due to insufficient recovery after thawing or suboptimal stimulation conditions. Allow thawed cells to recover 14-18 hours, ensure viability above 80% and optimize seeding density and nutrient supply. For polyclonal activation, confirm even coating of stimulation antibodies. Titrate PMA/Iono and BFA concentrations as well as stimulation times to balance activation strength with cell health, as all three components can be toxic to cells.

### Problem 5

Low tumor cell viability even without CAR T cells (e.g., during the Serial killing assay).

### Potential solution

Tumor cells may not tolerate the chosen medium, seeding density, or imaging conditions. Some tumor lines are sensitive to nutrient composition or serum levels. Test different medium formulations or combinations that support both tumor cells and CAR T cells. Adjust seeding density to maintain growth and morphology.

### Problem 6

Weak GFP detection in imaging software (e.g., during the Serial killing assay).

### Potential solution

GFP intensity can fluctuate during a serial killing assay due to changes in tumor-cell health, reporter stability, or imaging conditions, which can reduce automated detection accuracy. Always verify that the software correctly identifies GFP-positive tumor cells by comparing automated segmentation with manual inspection. If the software fails to detect tumor cells, alternative readouts such as GFP-positive cell count or overall GFP intensity can be used instead of GFP area to quantify tumor killing.

### Problem 7

Cell loss during washing steps (e.g., during the Preparation of single stained controls).

### Potential solution

Cells may detach or be lost due to harsh centrifugation, fragile post-fixation morphology, or disruption of the pellet during aspiration. To minimize this, adjust centrifugation speed by using lower g forces for live cells and slightly higher forces after fixation, and remove supernatant gently with a vacuum pump. Increasing wash volumes or switching to larger tubes may further reduce shear stress and improve overall cell recovery.

## Resource availability

### Lead contact

Further information and requests for resources and reagents should be directed to and will be fulfilled by the lead contact, Michael Schmueck-Henneresse (michael.henneresse@uhn.ca).

### Technical contact

Further information and requests of a technical nature should be directed to and will be fulfilled by the technical contact, Sarah Schulenberg (sarah.schulenberg@charite.de).

### Materials availability

This study did not generate new unique reagents. Patient-derived (C7R) GD2.CAR T cell products were obtained as part of the GAIL-B clinical trial (ClinicalTrials.gov identifier: NCT04099797) and are not available for distribution.

### Data and code availability

This paper does not report original code or new protein structural data. Datasets generated and analyzed in this study, including raw full spectrum flow cytometry files and source data for the figures, are available from the corresponding author upon reasonable request.

## Acknowledgments

We thank the patients and their families for participation in the study providing CAR T cell products. CAR T cell products were obtained through the GAIL-B clinical trial (ClinicalTrials.gov: NCT04099797) conducted at Baylor College of Medicine. This work was supported by the German Federal Ministry of Education and Research (10.13039/501100002347BMBF, CONAN grant 16GW0328K), the 10.13039/501100017268BIH Center for Regenerative Therapies, research grants from the 10.13039/501100015678Einstein Center for Regenerative Therapies, and funding from the European Rare Diseases Research Alliance (ERDERA) under the European Union’s Horizon Europe research and innovation programme (grant no. 101156595). The graphical abstract was created with BioRender.com. We would like to acknowledge the technical support of the 10.13039/501100017268BIH Cytometry Core Facility. We thank all voluntary blood donors for their donations.

## Author contributions

S.S. led the development and optimization of the protocol and wrote the manuscript. M.L. contributed to manuscript writing and protocol refinement. M.S.-H. conceived and supervised the project, provided conceptual guidance, and contributed to manuscript writing and critical revision.

## Declaration of interests

The authors declare no competing interests.

## Declaration of generative AI and AI-assisted technologies in the writing process

During the preparation of this work, the authors used Claude (Anthropic) to assist with phrasing of the manuscript. After using this tool, the authors reviewed and edited the content as needed and take full responsibility for the content of the publication.
